# Editorial: Community series in mental illness, culture, and society: Dealing with the COVID-19 pandemic - Volume IV

**DOI:** 10.3389/fpsyt.2023.1181772

**Published:** 2023-03-22

**Authors:** Renato de Filippis, Samer El Hayek, Mohammadreza Shalbafan

**Affiliations:** ^1^Psychiatry Unit, Department of Health Sciences, University Magna Graecia of Catanzaro, Catanzaro, Italy; ^2^Medical Department, Erada Center for Treatment and Rehab in Dubai, Dubai, United Arab Emirates; ^3^Mental Health Research Center, Psychosocial Health Research Institute, Department of Psychiatry, School of Medicine, Iran University of Medical Sciences, Tehran, Iran

**Keywords:** caregiver burden, pandemic uncertainty, psychological impact, public health, SARS-CoV-2, self-harm, social distancing, social psychiatry

The COVID-19 pandemic has had widespread consequences, affecting not only physical health but also mental health, social interactions, and economic stability (1–3). The lockdown and social distancing measures have intensified these issues, particularly for vulnerable individuals, also influencing the delivery of mental health interventions (4). Moreover, the impact of the pandemic has varied across different sociocultural groups, influenced by norms, values, and religions (5–7). This highlights the need to consider not only medical and scientific aspects but also the broader societal and cultural dynamics in addressing public health crises.

Following the previous three volumes of our Community Series Research Topic entitled “*Mental Illness, Culture, and Society: Dealing with the COVID-19 Pandemic*” (8–10), this forth volume features nine new papers that delve deeper into the intricate relationship between the pandemic, society and mental health. The papers included in this collection explore this connection in various ways, highlighting how unique cultures, societies, and backgrounds around the world mediate this complex interplay (11).

Liu Z. et al. analyzed the prolonged effect of the pandemic, 3 years after the outbreak, through the impact of pandemic uncertainty on depression, pandemic preventive behavior intentions, and positive life attitudes The authors used convenient sampling to collect data from 530 participants and discovered that the role of the grouping variable was significant in moderating the impact of uncertainty on positive attitudes and intentions toward pandemic prevention behavior, but it did not have a significant effect on depression. These results revealed a non-linear relationship between pandemic uncertainty and pandemic prevention behavior, as well as positive attitudes toward life, which sheds light on the non-linear nature of the relationship between psychological characteristics and the pandemic.

Along the same lines, Peng et al. focused on the long-term effects of the pandemic by investigating the mental health condition of 1,014 patients from two large Fangcang shelter hospitals in Shanghai. The authors found that dysfunctional beliefs about sleep significantly increased anxiety, depression, and insomnia, particularly in females aged 18–40 years old, with lower education level, higher income, white-collar jobs, or those who believed that the pandemic would have severe economic consequences. Another larger study looking at data from 6,218 individuals at the Fangcang shelter hospital in Shanghai (3.57% of all admitted patients) was conducted by Yu et al. In an attempt to identify the risk factors associated with psychiatric drug use in patients infected with the Omicron variant, the authors discovered that most patients had no previous psychiatric disorders and were prescribed psychiatric medications for the first time. Findings also revealed that female patients, those who were unvaccinated, older individuals, those with longer hospital stays, and those with multiple comorbidities were at higher risk, independent of medication use.

Another relevant aspect, analyzed in the work of Nooraeen et al., was the increase in relapse of individuals with severe mental illness and the consequent burden on their caregivers following virus containment measures. In this article, the authors evaluated 86 psychiatric patients and their caregivers during three different pandemic waves, and 3 and 6 months after the last one. The pandemic has had a dramatic effect on both the relapse and hospitalization rate, with psychopathological aggravation and worsening of caregivers' condition.

Mao et al. applied the Health Belief Model on social networks to analyze COVID-19-related tweets published by national health departments of the United States, South Korea, the United Kingdom, Japan, Germany, and India. Results showed a homogenization in the health departments promotion strategies across countries as well as in the promoted health measures, while there were some differences among users' responses to such promotions.

Among the cognitive fallout of the pandemic in the general population, the work published by Rocha et al. focused on possible changes in the decision-making style caused by anxious and post-traumatic symptoms. Results revealed that individuals with higher trait anxiety were less likely to use rationality in decision-making, particularly when post-traumatic stress symptoms were more severe. Conversely, individuals with lower trait anxiety tended to rely more on reason-based decision-making when faced with higher levels of post-traumatic stress symptoms.

Rammouz et al. investigated how religion may work as a coping strategy for mental health disorders in a population of nursing and medical students in Morocco. In this cross-sectional study, although students without depression showed a higher level of religiosity than those with depression, multivariate regression analysis revealed that religiosity was not a significant factor, either as a risk or protective factor, for depression.

A relevant but often overlooked issue is the increase in self-harm among adolescents with mental health problems during COVID-19 related society-wide isolation. This topic was addressed by the group led by Liu W. et al. Screening 63,877 medical records of children and adolescents who visited the Shanghai Mental Health Center in China between 2017 and 2021, authors demonstrated a global significant increase in self-harm rate in the past 5 years, with a peak in female patients aged between 12 and 13 years, especially among those with emotional disorders, during COVID-19 lockdown measures.

Finally, we report twelve naturalistic cases of first-episode mania after COVID-19 onset in the paper by Saeidi et al. Patients with a family history of mood disorders experienced a shorter onset of mania, whereas there was no significant difference in those who received corticosteroids. Although these results are anecdotal, they nonetheless identify a new line of research with potential clinical implications.

In summary, the articles described in the fourth volume of this Research Topic highlight the ongoing importance of cultural and social factors in shaping the psychiatric consequences of the coronavirus outbreak. Despite the newfound interest in social psychiatry evidenced by the data collected in our Community Series, the COVID-19 pandemic has highlighted the vulnerabilities experienced by the most fragile segments of society and the need for further clinical and epidemiological research in this field going forward.

## Author contributions

All authors listed have made a substantial, direct, and intellectual contribution to the work and approved it for publication.
